# Gene activation in human cells using CRISPR/Cpf1-p300 and CRISPR/Cpf1-SunTag systems

**DOI:** 10.1007/s13238-017-0491-6

**Published:** 2017-11-21

**Authors:** Xin Zhang, Wei Wang, Lin Shan, Le Han, Shufeng Ma, Yan Zhang, Bingtao Hao, Ying Lin, Zhili Rong

**Affiliations:** Cancer Research Institute, School of Basic Medical Sciences, Southern Medical University, Guangzhou, 510515 China

**Dear Editor,**

Clustered regularly interspaced short palindromic repeats (CRISPR) system is part of the adaptive immunity of bacteria and archaea that defends them against phage infection (Barrangou and Doudna, 2016). Variant CRISPR systems have been identified and harnessed for a wide range of applications in various organisms, exemplified by gene editing with the CRISPR/Cas9 system from *Streptococcus pyogenes* (Barrangou and Doudna, 2016). CRISPR/Cpf1 is a class 2 type V CRISPR system that differs from the counterpart CRISPR/Cas9 system. Cpf1 processes precursor CRISPR RNA (crRNA) by itself, uses a single crRNA to recognize a T-rich protospacer-adjacent motif (PAM) and finally induces sticky ends (Fonfara et al., 2016; Zetsche et al., 2015). The unique properties make CRISPR/Cpf1 a promising gene editing tool and a potential alternative to CRISPR/Cas9. Cpf1 has been used for effective genome editing in plants (Zaidi et al., 2017), mice (Kim et al., 2016) and human cells (Zetsche et al., 2015). Engineered dCpf1-SRDX, DNase dead Cpf1 fused to three copies of the SRDX transcriptional repressor, have been utilized to repress gene expression in *Arabidopsis* (Tang et al., 2017). Since Cpf1 is able to process its own crRNA (Fonfara et al., 2016), simultaneous multiplex gene editing using a single customized CRISPR array has been achieved (Zetsche et al., 2017). Furthermore, Cpf1 has advanced to correct disease-causing mutations in Duchenne muscular dystrophy (DMD) patient-derived induced pluripotent stem cells (iPSCs) and *mdx* mice, an animal model of DMD (Zhang et al., 2017). However, whether Cpf1 could be employed to activate gene expression remains unknown.

To repurpose Cpf1 as a transcriptional activator, the DNase activity of two commonly used Cpf1 from *Acidaminococcus sp*. *BV3L6* (AsCpf1) and *Lachnospiraceae bacterium ND2006* (LbCpf1) were deactivated via mutagenesis according to nuclease domain conservation (Zetsche et al., 2015), generating dLbCpf1 (D832A), dLbCpf1 (E925A), dLbCpf1 (D832A, D925A), and dAsCpf1 (D908A), dAsCpf1 (E993A), dAsCpf1 (D908A, E993A). Schematic of LbCpf1 and AsCpf1 DNase-dead mutants was shown in Fig. S1A. To verify deactivation of DNase activity, DNMT1 site 3 was selected as target site since this site has been successfully mutated with LbCpf1 and AsCpf1 in human cells (Zetsche et al., 2015). The polyacrylamide gel electrophoresis-based (PAGE) assay is a sensitive method to detect insertion or deletion mutations (indels) in mice and human cells, which is of higher sensitivity than T7 endonuclease I (T7E1) mismatch cleavage assay (Zhu et al., 2014). In PAGE assay, wild-type LbCpf1 and AsCpf1 demonstrated efficient DNA cleavage activity as evidenced by obvious various slowly-migrated heteroduplexes, while each mutant showed no DNase activity (Fig. S1B). Similar results were observed in T7E1 assay (Fig. S1C). All the above results demonstrated that variant DNase-dead Cpf1 mutants were successfully constructed.

As CRISPR/Cas9-based acetyltransferase could activate transcription (Hilton et al., 2015), we hypothesized that dCpf1 fused with a histone acetyltransferase would activate endogenous genes in a similar way. The histone acetyltransferase (HAT) core domain of human p300 (p300core, amino acids 1,048–1,664), containing bromodomain, CH2 region and HAT domain, retains inherent HAT activity (Delvecchio et al., 2013). Therefore, we fused dCpf1 with p300core and tested their capability to activate genes. Schematic of the fusion protein dLbCpf1-p300core was shown in Fig. 1A. Western blotting showed that all the three dLbCpf1-p300core fusion proteins were expressed at an even level (Fig. 1B). These fusion proteins, dLbCpf1 (D832A)-p300core, dLbCpf1 (E925A)-p300core, and dLbCpf1 (D832A, E925A)-p300core, were termed as M832, M925 and DM (double-mutation), respectively.

**Figure 1:**
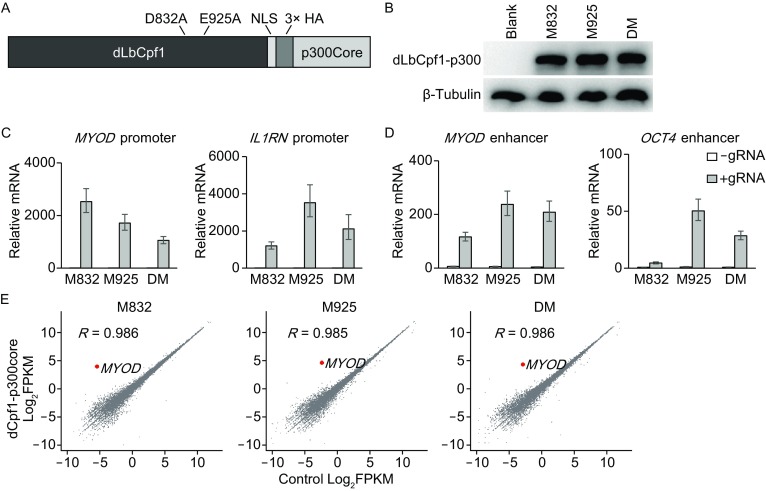
dLbCpf1-p300core fusion proteins activate endogenous gene expression from promoters and enhancers with high specificity. (A) Schematic of dLbCpf1-p300core fusion protein. p300core is the catalytic core of human p300 consisting of bromodomain, CH2 region and histone acetyltransferase (HAT) domain. (B) Western blot of dCpf1-p300core fusion proteins in transfected HEK293T cells. (C) Relative mRNA expression of *MYOD* and *IL1RN*, revealed by quantitative real-time PCR, in HEK293T cells co-transfected with dCpf1-p300core fusion proteins and four gRNAs targeting each promoter region of target genes. Mean value are presented with S.D. (*n* = 3). (D) Relative mRNA expression of *MYOD* and *OCT4* in HEK293T cells co-transfected with dCpf1-p300core fusion proteins and four gRNAs targeting the enhancer region. Mean value are presented with S.D. (*n* = 3). For (C) and (D), Tukey-test, *P* < 0.05 compared to cells transfected with dCpf1-p300core only, *n* = 3 independent experiments. (E) Gene expression plots generated from genome-wide RNA-seq data from HEK293T cells transiently co-transfected with dCpf1-p300core fusion proteins and four gRNAs targeting *MYOD* promoter compared to HEK293T cells transiently transfected with the corresponding dLbCpf1-p300core expression plasmid only. *MYOD* mRNAs are illustrated in red dots. *R* indicates Pearson’s correlation coefficient, calculated for log-transformed values on all genes except *MYOD*. Genes with 0 FPKM in either the activation sample or the control were excluded before log transformation

When each dLbCpf1-p300core fusion protein was co-transfected into HEK293T cells with four gRNAs targeting the respective promoter region of *MYOD* (also known as *MYOD1*) and *IL1RN*, the level of mRNA expression of *MYOD* and *IL1RN* was significantly increased (*P* < 0.05, Fig. 1C). However, only marginal activation was observed in dAsCpf1-p300core transfected cells (data not shown). Therefore, only dLbCpf1-p300core proteins were used in the following experiments. To explore whether dLbCpf1-p300core proteins could induce transcription in cells derived from human tissues other than fetal kidney (the tissue origin of HEK293T cells), the system was tested in MCF7 and U2OS cells, a human breast cancer cell line and a human osteosarcoma cell line, respectively. As shown in Figs. S2A and 2B, *MYOD* and *IL1RN* were activated in both cell lines.

Since p300 can interact with active enhancers in addition to promoters (Rada-Iglesias et al., 2011), we hypothesized that dLbCpf1-p300core would activate genes when targeted to appropriate enhancers. HEK293T cells were co-transfected with dLbCpf1-p300core proteins and four gRNAs targeting the distal regulatory region (DRR), an enhancer about 5 kb upstream of *MYOD* gene. Compared to the dLbCpf1-p300core-transfected-only control, the mRNA expression level of *MYOD* was significantly induced in a quantitative real-time PCR assay (*P* < 0.05, Fig. 1D). In addition, the proximal enhancer about 1.25 kb upstream of *OCT4* gene was targeted with four gRNAs. Each dLbCpf1-p300core protein activated transcription significantly although with different efficiencies (*P* < 0.05, Fig. 1D). To test whether the enhancer-targeting strategy could function in other lineages, MCF7 and U2OS cells were transfected. However, none of the dLbCpf1-p300core proteins was able to significantly induce the expression of *MYOD* or *OCT4* when targeted to the same sites with the same gRNAs in either cell lines (data not shown). Therefore, transcriptional activation by enhancer-targeting dLbCpf1-p300core proteins could be achieved in a cell context dependent manner.

To assess the transcriptional activation specificity of dLbCpf1-p300core proteins, genome-wide RNA expression profile was analyzed with RNA-seq assay in HEK293T cells co-transfected with four *MYOD*-targeted gRNAs and either dLbCpf1 (D832A)-p300core, dLbCpf1 (E925A)-p300core, or dLbCpf1 (D832A, E925A)-p300core. Gene expression differences were compared between dLbCpf1-p300core/gRNA-co-transfected cells and corresponding dLbCpf1-p300core-transfected-only controls. Gene expression was not broadly affected by either dLbCpf1-p300core protein (Pearson’s correlation coefficient *R* = 0.986, 0.985, and 0.986 for M832, M925 and DM, respectively; Fig. 1E). Among the genes with FPKM > 0, |log_2_Ratio| ≥ 1 and q-value | FDR ≤ 0.05, *MYOD* was the most highly upregulated gene in either fusion protein transfected cells (Fig. S3), indicating that the dCpf1 approach was robust and specific.

To test whether dLbCpf1-p300core proteins could be used to simultaneously activate multiple human genes with a minimal number of gRNAs, we examined the capability of single gRNA to activate gene. As shown in Fig. 2A, *MYOD*, *IL1RN*, and *HBG2* were activated with different efficiency using four single gRNAs for each gene promoter. No additive or synergistic effect was observed. The best single gRNAs showed higher activation efficiency than pooled single RNAs for each of the tested three genes. In addition, single gRNAs targeting the enhancer region (HS2) of *HBG2* showed similar results though with lower efficiency than that targeting promoter region (Fig. 2B). The best single gRNAs for *MYOD*, *IL1RN* and *HBG2* were pooled together and co-transfected into HEK293T cells with dLbCpf1-p300core proteins. As shown in Fig. 2C, each of dLbCpf1 (M832)-p300core, dLbCpf1 (M925)-p300core, and dLbCpf1 (DM)-p300core was able to simultaneously activate all the three genes, and the activation efficiency of pooled gRNAs was comparable to that of single individually gRNAs.

**Figure 2:**
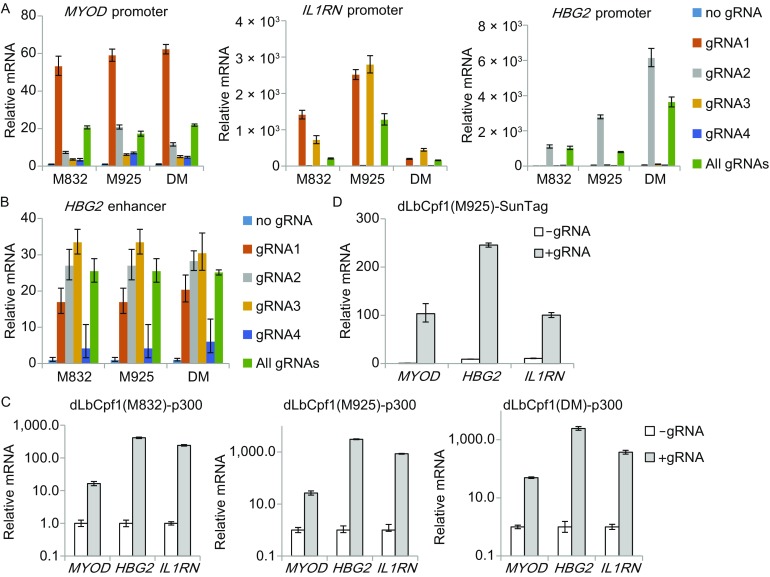
Simultaneously transcriptional activation of multiple endogenous genes using either dLbCpf1-p300core or dLbCpf1-SunTag system with a single gRNA for each gene. (A) Relative mRNA expression of *MYOD*, *IL1RN*, and *HBG2* revealed by quantitative real-time PCR, in HEK293T cells co-transfected with dCpf1-p300core fusion proteins and four single gRNAs or pooled sets of all four single gRNAs targeting each promoter region of target genes. (B) Relative mRNA expression of *HBG2* revealed by quantitative RT-PCR, in HEK293T cells co-transfected with dCpf1-p300core fusion proteins and four single gRNAs or pooled sets of all four single gRNAs targeting the enhancer region (HS2 region) of *HBG2* gene. (C) Relative mRNA expression of *MYOD*, *HBG2*, and *IL1RN* revealed by quantitative RT-PCR, in HEK293T cells co-transfected with dCpf1-p300core fusion proteins and three gRNAs targeting each promoter region of target genes. (D) Relative mRNA expression of *MYOD*, *HBG2*, and *IL1RN* revealed by quantitative RT-PCR, in HEK293T cells co-transfected with dLbCpf1 (M925)-SunTag and three gRNAs targeting each promoter region of target genes. For C and D, gRNA1, gRNA2 and gRNA1 were used for *MYOD*, *HBG2* and *IL1RN*, respectively. For (A–D), mean value are presented with S.D. (*n* = 3). Tukey-test, *P* < 0.05 compared to cells transfected with dCpf1-p300core or dLbCpf1(M925)-SunTag only, *n* = 3 independent experiments

SunTag system is a protein-tagging system, which consists of an array of repeating peptide and a single-chain variable fragment (scFv) antibody-fusion protein, which can bind to each other. This system has been successfully applied in live imaging and gene regulation (Tanenbaum et al., 2014; Ye et al., 2017). Therefore, we fused the GCN4 peptide repeat (10X) to the C-terminus of dLbCpf1 (M925), and fused the transcription factor VP64 to the GCN4 scFv antibody. When the two plasmids were co-transfected into HEK293T cells with the best single gRNAs for *MYOD*, *IL1RN*, and *HBG2* genes, all the three genes were simultaneously activated (Fig. 2D).

In this study, we developed dCpf1-p300core proteins to activate transcription from either promoters or enhancers in human cells. This approach is robust, specific, and functional in variant lineage cells. Furthermore, dCpf1-p300core proteins and dCpf1-SunTag system are able to simultaneously activate expression of multiple genes with a single gRNA targeting each gene. In addition, engineered Cpf1 variants with altered PAM specificities might replace wild type Cpf1, which can increase the targeting range of Cpf1-mediated genome regulation (Gao et al., 2017). Therefore, Cpf1 is a promising alternative to Cas9, and Cpf1-based approaches hold the potential to be developed into a versatile gene editing toolbox, which would greatly expand the applicability of CRISPR system in genome editing.

## Footnotes

We thank Rong and Lin lab members for constructive criticism and discussions. The research was funded by the National Natural Science Foundation of China (Grant Nos. 81670093 and 81372494), the Natural Science Foundation of Guangdong Province (2014B020212018 and 2017A030310331), the Program of Guangzhou Science Technology and Innovation Commission (201508020120), and the Thousand Young Talents Program of China.

Xin Zhang, Wei Wang, Lin Shan, Le Han, Shufeng Ma, Yan Zhang, Bingtao Hao, Ying Lin and Zhili Rong declare that they have no conflict of interest.

## Electronic supplementary material

Below is the link to the electronic supplementary material.

Supplementary material 1 (PDF 421 kb)
