# Correction to: Gene activation in human cells using CRISPR/Cpf1-p300 and CRISPR/Cpf1-SunTag systems

**DOI:** 10.1007/s13238-018-0585-9

**Published:** 2018-11-12

**Authors:** Xin Zhang, Wei Wang, Lin Shan, Le Han, Shufeng Ma, Yan Zhang, Bingtao Hao, Ying Lin, Zhili Rong

**Affiliations:** Cancer Research Institute, School of Basic Medical Sciences, Southern Medical University, Guangzhou, 510515 China

## Correction to: Protein Cell 2018, 9(4):380–383 10.1007/s13238-017-0491-6

In the original publication the Supplementary Material and Fig. 2 are incorrect. The correct version of Supplementary Material and Fig. 2 are provided in this correction article. The text HBG2 appearing in the article should be read as HBG1.

**Figure 2:**
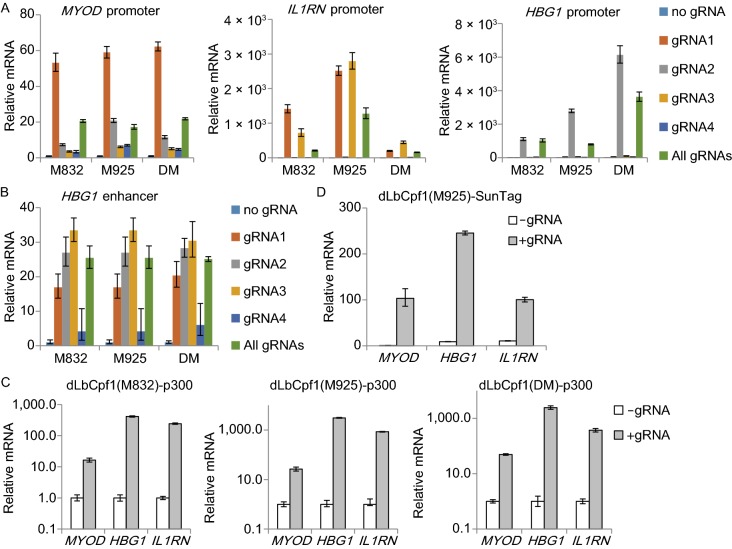
**Simultaneously transcriptional activation of multiple endogenous genes using either dLbCpf1-p300core or dLbCpf1-SunTag system with a single gRNA for each gene**. (A) Relative mRNA expression of *MYOD*, *IL1RN*, and *HBG1* revealed by quantitative real-time PCR, in HEK293Tcells co-transfected with dCpf1-p300core fusion proteins and four single gRNAs or pooled sets of all four single gRNAs targeting each promoter region of target genes. (B) Relative mRNA expression of *HBG1* revealed by quantitative RT-PCR, in HEK293T cells co-transfected with dCpf1-p300core fusion proteins and four single gRNAs or pooled sets of all four single gRNAs targeting the enhancer region (HS2 region) of *HBG1* gene. (C) Relative mRNA expression of *MYOD*, *HBG1*, and *IL1RN* revealed by quantitative RT-PCR, in HEK293Tcells co-transfected with dCpf1-p300core fusion proteins and three gRNAs targeting each promoter region of target genes. (D) Relative mRNA expression of *MYOD*, *HBG1*, and *IL1RN* revealed by quantitative RT-PCR, in HEK293T cells co-transfected with dLbCpf1 (M925)-SunTag and three gRNAs targeting each promoter region of target genes. For C and D, gRNA1, gRNA2 and gRNA1 were used for *MYOD*, *HBG1* and *IL1RN*, respectively. For (A–D), mean value are presented with S.D. (*n* = 3). Tukey-test, *P* < 0.05 compared to cells transfected with dCpf1-p300core or dLbCpf1(M925)-SunTag only, *n* = 3 independent experiments

## Electronic supplementary material

Below is the link to the electronic supplementary material.

Supplementary material 1 (PDF 677 kb)

