# Supplementary material for: Correction to: Gene activation in human cells using CRISPR/Cpf1-p300 and CRISPR/Cpf1-SunTag systems
Source: Protein Cell. 2018 Nov 12;10(10):776–7. doi: 10.1007/s13238-018-0585-9 (PMC6776487; doi:10.1007/s13238-018-0585-9)
Supplement: Supplementary file 1 — Supplementary material 1 (PDF 677 kb) [file 13238_2018_585_MOESM1_ESM.pdf]

Supplementary Methods

Plasmids Construction

The DNA sequences of human codon-optimized AsCpf1 and LbCpf1 with NLS and HA tag were synthesized from iGene. DNase deactivated mutants were constructed by mutagenesis with Gibson Assembly. The sequence of human p300core was amplified from HEK293T cells and sub-cloned into dCpf1 expression plasmids. Cpf1, dCpf1 and dCpf1-p300core were sub-cloned into a mammalian expression vector containing a pBlueScript backbone, a CAG promoter and a bGH poly A signal sequence. To generate SunTag system plasmid, the 10xGCN4 sequence amplified from pHRdSV40-dCas9- 10xGCN4_v4-P2A-BFP plasmid (Addgene #60903) was fused to dCpf1 expression vector by ligation. And the scFv-GCN4-sfGFP-VP64-GB1-NLS from pHRdSV40-scFv-GCN4-sfGFP-VP64-GB1-NLS plasmid (Addgene #60904) was cloned into a pBlueScript backbone with an EF-1-alpha core promoter and a SV40 polyA signal sequence. To generate gRNA expression plasmid, pBlueScript SK+ vector and the U6 promoter sequence amplified from pLentiCRISPR v2 plasmid (Addgene #52961) via PCR were fused by Gibson Assembly. Annealed oligos for AsCpf1 or LbCpf1 gRNA scaffold were ligated to the pBlueScript-U6 vector to generate gRNA basic plasmids. gRNA expression plasmid for each target site was constructed by ligating the annealed corresponding oligos to the basic plasmid. gRNA oligos were designed through *www.Benchling.com*. Primers were listed in Table 1.

Cell lines and transfection

HEK293T and U2OS cells were cultured in Dulbecco's modified Eagle's medium (DMEM) supplemented with 10% FBS and 1% penicillin/streptomycin and maintained at 37 °C and 5% CO2. MCF7 cells were cultured in RPMI 1640 medium supplemented with 10% FBS and 1% penicillin/streptomycin and maintained at 37 °C and 5% CO2. Transfections were performed in 12-well plates using 200ng of respective dCpf1-p300core expression plasmids and 800ng of equimolar pooled gRNA expression vectors with polyethylenimine (PEI) solution. 48 hr later, genomic DNA was harvested for PAGE and T7E1 assays, or total RNA was harvested for gene expression assay.

Polyacrylamide gel electrophoresis (PAGE) assay

Genomic DNA was isolated with sarkosyl lysis buffer (10 mM Tris pH7.6, 10 mM EDTA, 10 mM NaCl, 0.5% Sarkosyl, 0.1 mg/ml proteinase K) and amplified by PCR with primers flanking target sites. After reannealed to form heteroduplexs, PCR amplicons were subjected to 5% polyacrylamide gel electrophoresis and stained in 2xGel-red solution before ultraviolet imaging.

T7 endonuclease I (T7E1) assay

PCR amplicons containing target genomic region were purified with Qiaquick PCR Purification Kit (Qiagen). Purified PCR products were denatured and cooled down to form heteroduplex DNA in NEB buffer 2 using a thermocycler. Annealed PCR amplicons were incubated with T7 endonuclease I (NEB) for 90min at 37 °C and

subjected to 2% agarose gel electrophoresis.

Western blotting

The HEK293T cells transiently transfected with dCpf1-p300core plasmids were lysed in 2XSDS loading buffer (100 mM Tris pH 6.8, 4% SDS, 20% glycerol, 200 mM β-mercaptoethanol, 0.05% bromophenol blue) and boiled for 10 min. The lysates were resolved by SDS-PAGE and transferred onto a nitrocellulose membrane. The membrane was blocked with 5% non-fat milk in TBST containing 0.05% Tween 20, and sequencially incubated with the indicated primary antibodies (anti-HA, MBL m180-3; anti-β-tubulin, Proteintech 66240-I-Ig) and HRP-conjugated horse anti-mouse IgG secondary antibody (CST 7076S). The probed proteins were finally detected by chemiluminescence according to manufacturer's instructions (Pierce)

Quantitative real-time PCR

Total RNA was isolated from transfected cells with RNeasy Mini Kit (Qiagen) and reverse transcripted into cDNA using PrimeScript RT Reagent Kit (TAKARA). Quantitative real-time PCR was performed using SYBR Premix Ex Taq II (TAKARA) with a Mx3005p Thermal Cycler (Agilent Technologies). Results are expressed as fold change above mock-transfected cells after normalizing to GAPDH expression using the ΔΔCt method. All qPCR primers are listed in Table 1.

RNA-seq analysis

Total RNA was isolated from transfected HEK293T cells with RNeasy Mini Kit

(Qiagen). Briefly, mRNA was enriched and fragmentated. And random hexamers were used for the first strand synthesis, followed by the second strand synthesis. After cleaned up, double strand cDNA was added with adaptors and amplified by PCR to finanize library construction. After verified using Qubit 3.0, Agilent 2100 Bioanalyzer and quantitative RT-PCR, libraries were pooled and sequenced on an Illumina HiSeq X Ten with 150bp paired-end module.. Reads were aligned to the hg19 transcriptome using Tophat2, and gene expression was quantified using Cufflinks 2.2.1. Differential expression was defined by a Benjamini-Hochberg adjusted p value (q value | FDR) of <0.05 and fold change of >2 or <0.5.

Supplementary Figure Legends

Figure S1. Generation of LbCpf1 and AsCpf1 mutants without DNase activity. (A) Schematic of LbCpf1 and AsCpf1 DNase-dead mutants. dLbCpf1 mutants contain DNase-deactivating mutations D832A, E925A or both, while dAsCpf1 mutants contain D908A, E993A or both. NLS, nuclear localization signal; HA, human hemagglutinin epitope tag. (B and C) PAGE and T7E1 analyses showing the DNase activities of wide-type and mutants of Cpf1 in human cells. DNA samples were harvested from HEK293T cells co-transfected with wide-type or mutants of Cpf1 and DNMT1 site 3 targeted gRNA plasmids.

Figure S2. dLbCpf1-p300core proteins activate gene expression in multiple cell lines. (A & B) Relative mRNA expression of *MYOD* and *IL1RN* in U2OS cells (A) and MCF7 cells (B) co-transfected with dCpf1-p300core fusion proteins and four gRNAs targeting each promoter region of target genes. Mean value are presented with S.D. (n = 3). Tukey-test, $p < 0.05$ compared to cells transfected with dCpf1-p300core only, n = 3 independent experiments.

Figure S3. Transcriptional activation by dLbCpf1-p300core fusion proteins is specific and robust. Histograms of the distribution of fold changes in gene expression (Log2 (activation /control)). The Histograms were generated from RNA-seq data from HEK293T cells transiently co-transfected with dLbCpf1-p300core proteins and four gRNAs targeting *MYOD* promoter. The control samples were transfected with dLbCpf1-p300core plasmids only. Genes with 0 FPKM were

excluded. The fold change of *MYOD* is indicated.

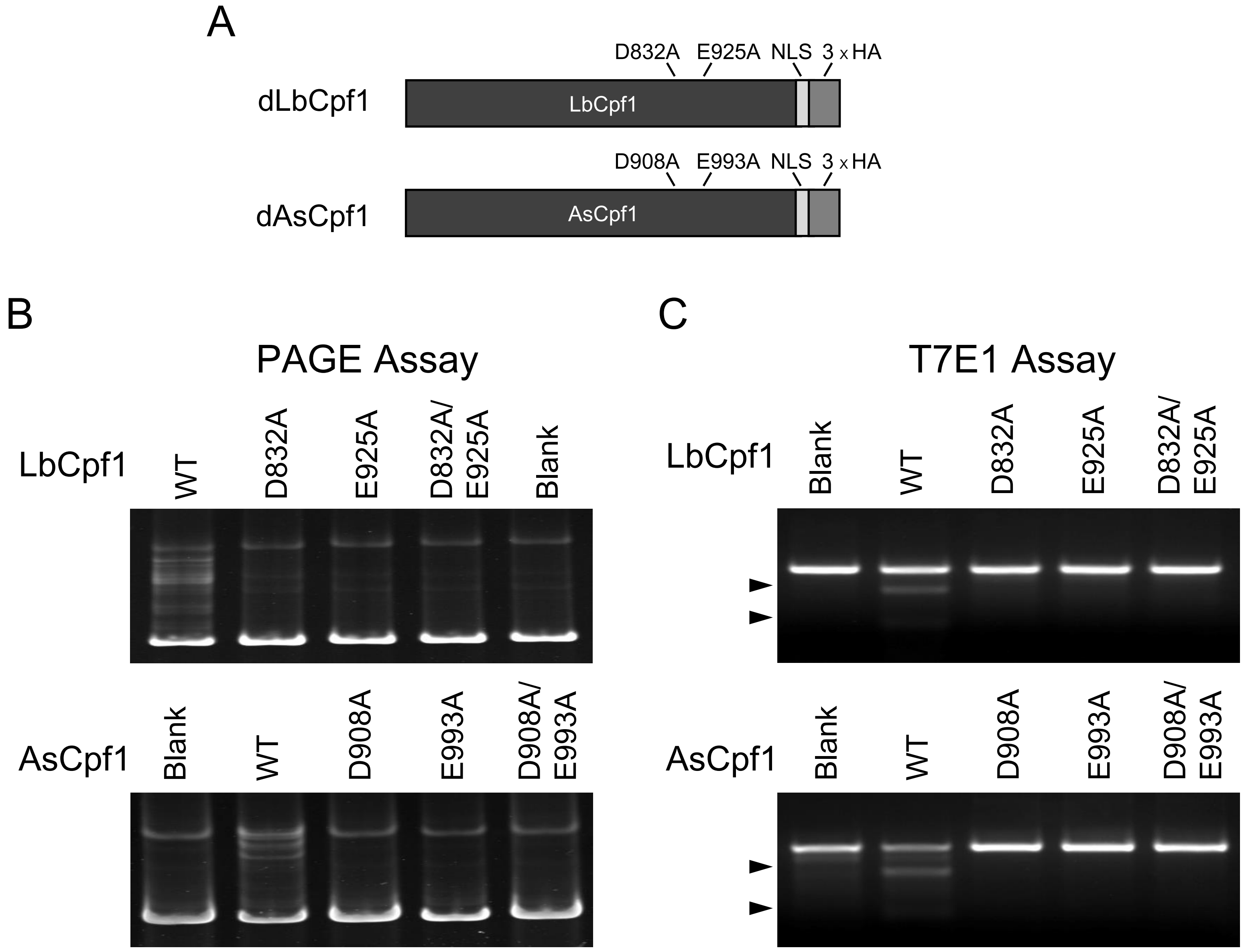
A
D832A
E925A
NLS
3 x HA
dLbCpf1
LbCpf1
D908A
E993A
NLS
3 x HA
dAsCpf1
AsCpf1
B
PAGE Assay
LbCpf1
WT
D832A
E925A
D832A/ E925A
Blank
AsCpf1
Blank
WT
D908A
E993A
D908A/ E993A
C
T7E1 Assay
LbCpf1
Blank
WT
D832A
E925A
D832A/ E925A
AsCpf1
Blank
WT
D908A
E993A
D908A/ E993A

Figure S1

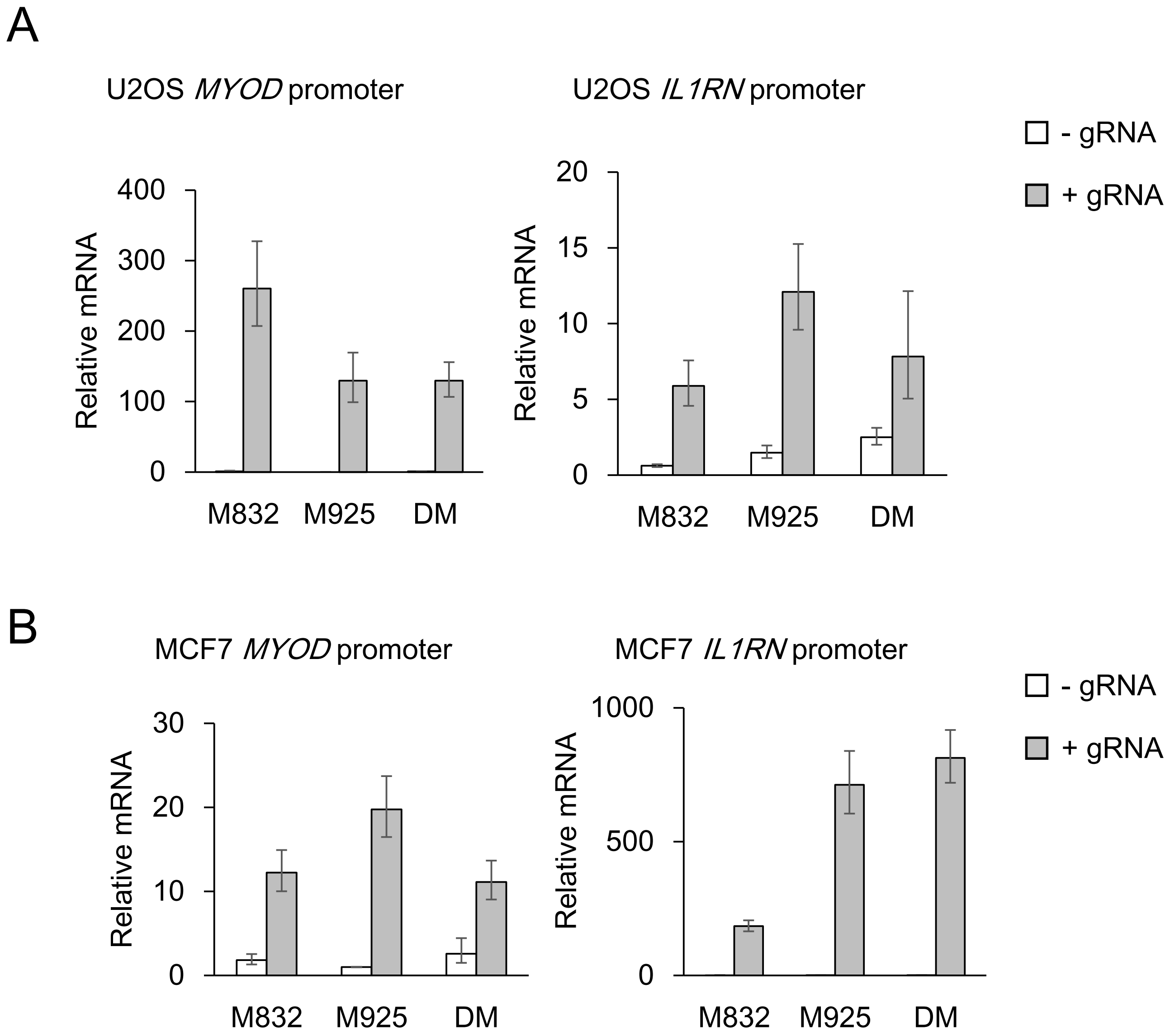
A
U2OS *MYOD* promoter
U2OS *IL1RN* promoter
- gRNA
+ gRNA
Relative mRNA
400
300
200
100
0
M832
M925
DM
Relative mRNA
20
15
10
5
0
M832
M925
DM
B
MCF7 *MYOD* promoter
MCF7 *IL1RN* promoter
- gRNA
+ gRNA
Relative mRNA
30
20
10
0
M832
M925
DM
Relative mRNA
1000
500
0
M832
M925
DM

Figure S2

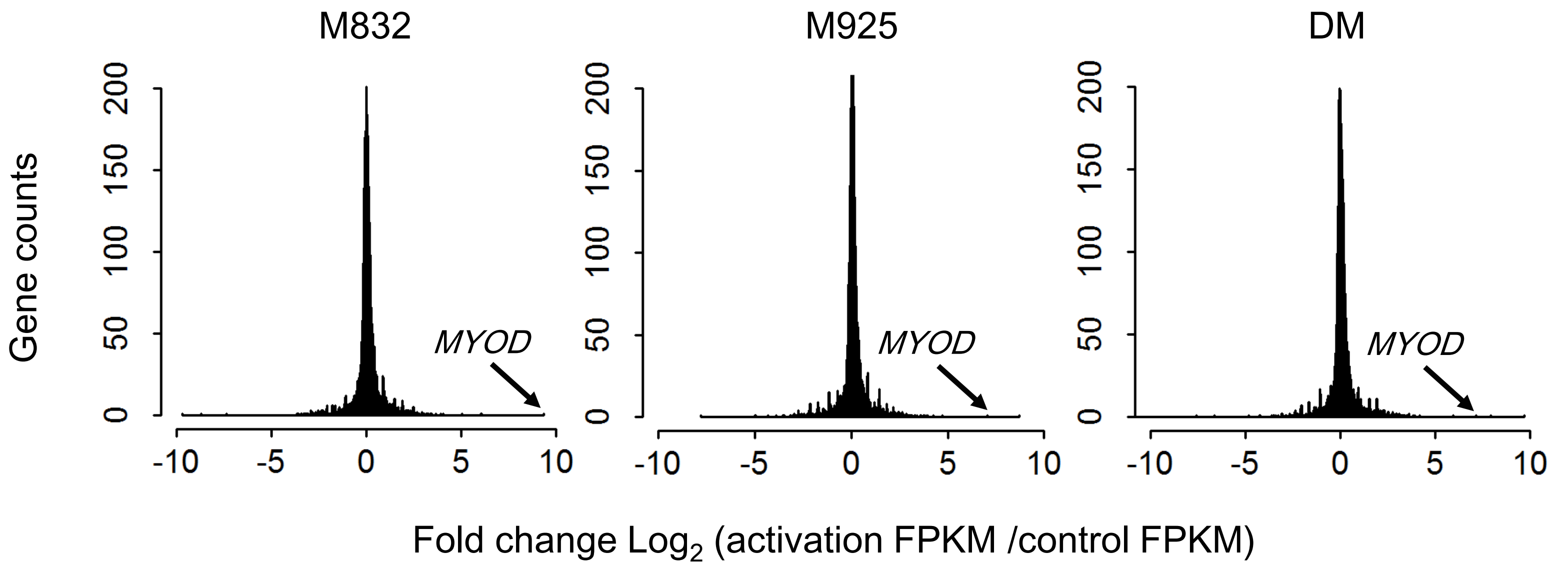

M832
M925
DM
Gene counts
0
50
100
150
200
MYOD
-10
-5
0
5
10
Fold change Log2 (activation FPKM /control FPKM)

Figure S3

Table 1. Primer list

gRNA primers	Sequence (5'-3')
MyoD-P1-F	GGCTACTACGGATAAATAGCCCATTTTTTTC
MyoD-P1-R	CCGGGAAAAAAATGGGCTATTTATCCGTAGTAGCC
MyoD-P2-F	TCCGTAGTAGCCTAAACGCCCCGTTTTTTTC
MyoD-P2-R	CCGGGAAAAAAACGGGGCGTTTAGGCTACTACGGA
MyoD-P3-F	GAAAGGGCGTGCCGGAGAGCCAATTTTTTTC
MyoD-P3-R	CCGGGAAAAAAATTGGCTCTCCGGCACGCCCTTTC
MyoD-P4-F	CCGCGGATACAGCAGTCGGGTGTTTTTTTTC
MyoD-P4-R	CCGGGAAAAAAAACACCCGACTGCTGTATCCGCGG
MyoD-DRR1-F	GCGCCCCCCACCTCCCGGCCAGATTTTTTTC
MyoD-DRR1-R	CCGGGAAAAAAATCTGGCCGGGAGGTGGGGGGCGC
MyoD-DRR2-F	TATATATAGCCTCTGGAAACCCATTTTTTTC
MyoD-DRR2-R	CCGGGAAAAAAATGGGTTTCCAGAGGCTATATATA
MyoD-DRR3-F	CCAGGGAGCAAGTTTGTCAGGGGTTTTTTTC
MyoD-DRR3-R	CCGGGAAAAAAACCCCTGACAAACTTGCTCCCTGG
MyoD-DRR4-F	CAGAGGCCAGCTCTCCATTTATATTTTTTTC
MyoD-DRR4-R	CCGGGAAAAAAATATAAATGGAGAGCTGGCCTCTG
IL1RN-P1-F	TTAGAGCGTTGGGGACCTTGTCTTTTTTTC
IL1RN-P1-R	CCGGGAAAAAAAGACAAGGTCCCCAACGCTCTAA
IL1RN-P2-F	GAATGAATGTGTGCACACATGCATTTTTTTC
IL1RN-P2-R	CCGGGAAAAAAATGCATGTGTGCACACATTCATTC
IL1RN-P3-F	CAGGAGGGTGACTCAGGCTAGCATTTTTTTC
IL1RN-P3-R	CCGGGAAAAAAATGCTAGCCTGAGTCACCCTCCTG
IL1RN-P4-F	TGCTAGCCTGAGTCACCCTCCTGTTTTTTTC
IL1RN-P4-R	CCGGGAAAAAAACAGGAGGGTGACTCAGGCTAGCA
OCT4-PE1-F	GGCCCCCTCCACTATGGAACCTGTTTTTTTC
OCT4-PE1-R	CCGGGAAAAAAACAGGTTCCATAGTGGAGGGGGCC
OCT4-PE2-F	GGGTTAGAGCTGCCCCCTCTGGGTTTTTTTC
OCT4-PE2-R	CCGGGAAAAAAACCCAGAGGGGGCAGCTCTAACCC
OCT4-PE3-F	GCGTCTCTGAAGGGGATTCTGTGTTTTTTTC
OCT4-PE3-R	CCGGGAAAAAAACACAGAATCCCCTTCAGAGACGC
OCT4-PE4-F	CCAACCTTTGCTGAAACAGAGTGTTTTTTTC
OCT4-PE4-R	CCGGGAAAAAAACACTCTGTTTCAGCAAAGGTTGG
DNMT1-3-F	CTGATGGTCCATGTCTGTTACTCTTTTT

DNMT1-3-R	GAGTAACAGACATGGACCATCAGATCTA
HBG1-P1-F	TCCTTAGAAACCACTGCTAACTGTTTTTTTG
HBG1-P1-R	GATCCAAAAAAAACAGAAATGTAACAGGAACTAAG
HBG1-P2-F	AACTACAGGCCTCACTGGAGCTATTTTTTTG
HBG1-P2-R	GATCCAAAAAAATAGCTCCAGTGAGGCCTGTAGTT
HBG1-P3-F	CCTTGTCAAGGCTATTGGTCAAGTTTTTTTG
HBG1-P3-R	GATCCAAAAAAACTTGACCAATAGCCTTGACAAGG
HBG1-P4-F	ATTCTTCATCCCTAGCCAGCCGCTTTTTTTG
HBG1-P4-R	GATCCAAAAAAAGCGGCTGGCTAGGGATGAAGAAT
HS2-1F	CTTAGTTCCTGTTACATTTCTGTTTTTTTTG
HS2-1R	GATCCAAAAAAAACAGAAATGTAACAGGAACTAAG
HS2-2F	CTTAGAAGGTTACACAGAACCAGTTTTTTTG
HS2-2R	GATCCAAAAAAACTGGTTCTGTGTAACCTTCTAAG
HS2-3F	TGCCATCTGCCCTGTAAGCATCCTTTTTTTG
HS2-3R	GATCCAAAAAAAGGATGCTTACAGGGCAGATGGCA
HS2-4F	GAAGCACCTGCTGGATGCTAAACTTTTTTTG
HS2-4R	GATCCAAAAAAAGTTTAGCATCCAGCAGGTGCTTC
**Mutation Primers**	**Sequence (5’-3’)**
AsCpf1-Mut-D908A-F	CGACCTGTCTGATGAGGCCAGGGCCCTGC
AsCpf1-Mut-D908A-Rm	CTCTCGCCCCGGGCGATGCCGATGATAGG
AsCpf1-Mut-D908A-Fm	ATCGGCATCGCCCGGGGCGAGAGAAAC
AsCpf1-Mut-D908A-R	AAAGTGGCACCGAGTCGGTGCGGATCC
AsCpf1-Mut-E993A-Rm	ATTCAGGTTGGCTAGCACCACCACGGC
AsCpf1-Mut-E993A-Fm	GGTGGTGCTAGCCAACCTGAATTTCGG
LbCpf1-Mut-D832A-Fm	AACCCCTATGTGATCGGCATCGCTAGGGGCGAGCGC
LbCpf1-Mut - R	CTTGAAAAAGTGGCACCGAGTCGGTGCGGATCC
LbCpf1-Mut-E925A-F	ACGATAACCCCTATGTGATCGGCATCGATAGG
LbCpf1-Mut-E925A-Rm	AGTTCAGGTCGGCTAGCGCGATCACGGCATCG

LbCpf1-Mut-E925A-Fm	CCGTGATCGCGCTAGCCGACCTGAACTCTGGC
**PCR Primers**	**Sequence (5’-3’)**
DNMT1-3-PCR-F	CAAGTGCTTAGAGCAGGCGT
DNMT1-3-PCR-R	GTGACGGGAGGGCAGAACTA
p300 PCR Primer-F	GCGCCTCGAGATTTTCAAACCAGAAG
p300 PCR Primer-R	ATATACCGGTGTCCTGGCTCTGCGTGT
10xGCN4 Primer-F	ATTATTACTCGAGAGCCTGGGCAGCGGCT
10xGCN4 Primer-R	GCCGTGTACATTACACCTTGCGCTTCTTC
**RT-PCR Primers**	**Sequence (5’-3’)**
GAPDH-qPCR-F	AGAAGGCTGGGGCTCATTTG
GAPDH-qPCR-R	AGGGGCCATCCACAGTCTTC
MyoD-qPCR-F	TCCCTCTTTCACGGTCTCAC
MyoD-qPCT-R	AACACCCGACTGCTGTATCC
IL1RN-qPCR-F	GGAATCCATGGAGGGAAGAT
IL1RN-qPCR-R	TGTTCTCGCTCAGGTCAGTG
OCT-4-qPCR-F	CGAAAGAGAAAGCGAACCAGTATCGAGAAC
OCT-4-qPCR-R	CGTTGTGCATAGTCGCTGCTTGATCGC
HBG1-qPCR-F	GCTGAGTGAACTGCACTGTGA
HBG1-qPCR-R	GAATTCTTTGCCGAAATGGA
